# Anatomy of the Teeth

**Published:** 1838-07-01

**Authors:** 


### ANATOMY OF THE TEETH.

I. ANATOMIE DU SYSTEME DENTAIRE, CONSIDÉRÉE DANS L'HOMME ET LES ANIMAUX. Par *Ph. Fr. Blandin*, Chirurgien de l'Hôtel Dieu, &c. Avec une Planche. 8vo. Pp. 232. Paris et Londres, 1836.

II. THE WORKS OF JOHN HUNTER, F.R.S., WITH NOTES. Edited by *James F. Palmer*, Senior Surgeon to the St. George's and St. James's Dispensary, &c. &c. In Four Volumes. Illustrated by a Volume of Plates, in quarto. Vol. II. on the Natural History and Diseases of the Human Teeth, &c. London, 1835.

THE principal object of the Treatise of Hunter is to describe the Diseases of the Human Teeth. Their natural history occupies a brief portion of it, and the comparative anatomy of these important organs is not treated of.

But the object of M. Blandin is at once more comprehensive and more limited—more comprehensive in all that relates to the Anatomy of the Teeth—more limited, because their diseases are not made the subject of examination.

There appear to be more valid reasons for dental surgery forming a sort of separate art, than for the surgery of the eyes or of the ears being made so. In the former, much mechanical contrivance is requisite, so much as to render it exceedingly improbable that any one but a special *artiste* could excel in it. But the operations necessary for the eyes and ears can be as well performed by a dexterous surgeon as by any oculist or aurist.

If, however, a surgeon is unlikely to make good artificial teeth, or to be au fait at the business of cleaning and stopping them, he ought to be well acquainted with their structure and their functions, and to be conversant with the laws of their development and their decay. This is an useful as well as a philosophical study, yet probably it is one on which a large number of medical men, are very scantily informed.

We shall present a pretty copious account of M. Blandin's Thesis, and introduce the more familiar sentiments of Mr. Hunter, when they seem to explain or to illustrate the text of the former author. We think we shall thus put our readers in possession of the most recent and most valuable information on a subject which cannot be uninteresting or unimportant to them.

M. Blandin observes, in his Preface, that he adopts the definition of Cuvier, and considers the teeth, as mechanical instruments harder than bone, and placed in the vertebrated animals at the entrance of the alimentary canal, to seize and comminute the food, or to serve as offensive or defensive weapons.

The plan which he pursues is briefly stated. He first takes a rapid survey of the history of the anatomy of the teeth—then he gives a sketch of the dental system in general—then carefully describes the human teeth—and concludes with a view of their varieties in the different classes of animals.

### I. History of the Anatomy of the Teeth.

In breaking this ground M. Blandin makes some eloquent complaints, which are true of more medical matters than the Anatomy of the Teeth. Every dentist, says he, has unfortunately thought it necessary to write his book, and "these repulsive compilations have succeeded each other with desperate pertinacity, from the infancy of science to the present day."—"A man must go like me," says M. Blandin, "the historical microscope in hand, on the hunt for the most minute discovery lost in the midst of an interminable heap of absurdities, to have any idea of the tardy progress of dental anatomy." In this, as well as in other cases, genuine discoveries have frequently met with the greatest opposition, and have been rejected for positive errors. Thus many modern novelties are of ancient date, and many a discovery has had many fathers. The child, indeed, has been often born before its parent, and would seem to have reversed the fable of Saturn, and devoured those who gave it birth.

M. Blandin divides the History of Dental Anatomy into epochs. We do not think it necessary for our present purpose to go into either one or the other, and shall merely note any curious or interesting circumstance that may be noticed, without respect to chronology.

*a.* M. Blandin cites, and we may quote, the definition of the teeth given by Homer:—"Barriers opposed by nature to slips of the tongue and the abuse of speech."

We know not how modern dentists will like the hint conveyed by the fact, that, in the temple of Apollo was suspended a tooth-instrument made of *lead*, to shew that only those teeth should be pulled out which could be removed by so weak an extractor.

*b.* Aristotle, that universal genius, actually asserts that men have more teeth than women, and that this is the case with several other animals, as sheep, goats, and pigs! Nothing can more decisively demonstrate the inexact philosophy of former times than such specimens of it as this. A man writes an elaborate treatise, in which he asserts ex cathedrâ as a fact, what the very slightest examination of matters daily under his nose would tell him in an instant was no fact at all.

*c.* Aretæus makes a curious and a candid confession. "God alone," says he, "knows the cause of the tooth-ache."

*d.* Pliny has his share of the wonderful. He assures us that human teeth contain a pernicious virus, and that a bite from them will destroy weak animals! So it would, no doubt, were it strong and they weak *enough*.

*e.* Sylvius said rather a good thing. Having copied the anatomical errors of Galen, and the discoveries of Vesalius, which exposed those errors, having been objected to him, he replied, that:—"Galen could not have been wrong, and that the human organization must have changed since his time."

*f.* Thomas Bartholin is often quoted as an anatomical authority. His value may, in some degree, be estimated from this circumstance. He states in his works that he saw a man in whose jaw there grew an iron tooth; and, what is more, he enters into an argument to show how such a thing may be!

M. Blandin pays moderate attention, and a respectable compliment to Hunter. We will not go so far as to say that he underrates the extent and importance of his labours, but certainly he does not over-estimate them. Whether the Preface of Mr. Bell to the present edition of John Hunter's works may be thought to do the latter, may perhaps admit of question.

"The state of Dental Surgery," says Mr. Bell, "at the period when Hunter wrote the following work was perhaps lower than that of any department of professional science or practice. The treatment of the teeth was still consigned to the hands of the ignorant mechanic, whose knowledge was limited to the forcible extraction of aching teeth, the manufacture of substitutes for those which were lost, and some rude methods of filling the cavities produced by decay. That this state of a branch of practice, as susceptible of a connexion with physiological and pathological science, and as improveable by such a connexion as any other, should have early attracted the attention of a man pre-eminently qualified for detecting and supplying such deficiencies, and whose labours, unparalleled as they are for their scientific importance, are not less valuable from the immense influence they have since exerted upon the practice both of medicine and surgery, might have been anticipated, from the peculiar character of his mind, which was too truly great to think any subject unworthy of his anxious attention which involved the improvement of the art of healing, or the extension of our knowledge of Nature's operations. If it may be stated that the work in question is perhaps the least felicitous effort of this extraordinary genius, and that of which the errors are the most obvious and striking, some apology may be found even for these, in the confined nature of the subject, and especially in the obscure and anomalous structure of the organs of which it treats; whilst the basis which his experiments and observations have laid for subsequent improvements in our knowledge, both of the physiology and pathology of the teeth as well as in the treatment of their diseases, constitutes a never-ceasing claim to the gratitude and admiration of every scientific practitioner of dental surgery. If, therefore, it may with truth be said that he was the father of scientific surgery; if he may claim the high distinction of having placed the practice of surgery upon the only solid foundation, that of physiological science, it is no less true of this humble department than of those more important branches of the art, in which are involved the knowledge and treatment of diseases which stand in immediate connexion with vital organs and functions." xii.

We pass over the remainder of M. Blandin's historical review of authors, and proceed to the Second Part of his Thesis, which opens with some observations on:—

### II. THE TEETH IN GENERAL.

He conceives that the most simple idea of teeth is to consider them as resisting bodies placed at or near the entrance of the alimentary canal, for the purpose of seizing and dividing the aliment, sometimes for the purposes of aggression or defence.

Teeth are a genuine production of the internal tegumentary system, of the mucous membrane which lodges them in a depression to which they adhere by one extremity. Sometimes they have no sort of connexion with the osseous system; most frequently they rest on it, either at its surface or in special cavities called *alveoli.*

In general the teeth are arranged in a row, or at all events, are so opposed to one another at their free extremity, as to constitute pincers more or less sharp or blunted.

The Dental Organs are essentially composed of two elements—the part secreting, and the part secreted.

The *part secreting*, the *Matrix, Follicle, Bulb*, or *Germ*, is an immediate dependence of the tegumentary system, a little sac analogous to the follicles of that system, with the exception, that, from its interior, there projects a *papilla* or *pulp* of variable form; a fasciculus of blood-vessels and nerves entering its base, unite it to the neighbouring parts; while its other extremity presents an opening, the *neck* of the follicle, which is closed before the eruption of the tooth, but at a little later period forms a *gubernaculum* to it.

The *part secreted* is the tooth. Its form varies exceedingly. It is composed of two parts—the crown and root—the former free, the latter implanted in the follicle and almost always hollowed to receive the *pulp*.

The *Follicle* has the membranous organization of the integuments in general, differing from them only in the growth of so considerable a papillary body from one point.

The teeth are formed of ossiform calcareous layers, included one within the other, and impermeable to the vessels and nerves of the Follicle.

The tooth is developed *within* the Follicle and *on* the Papilla by delicate layers which embrace it. The Papilla secretes these layers, each successive one of which is *within* its predecessors. The tooth elongates until it can no longer be contained within the follicle, when it penetrates it to make its way to the exterior. In doing so, sometimes it dilates the neck of the Follicle—sometimes it traverses it in a slightly different direction.

After its eruption, the tooth continues, for a longer or a shorter time, to increase, by the addition of fresh layers on the inside of the old. Then commences the process of destruction from without, the consequence of the attrition to which the tooth is exposed.

The duration of the teeth is limited by nature, who has in this manner limited also the duration of animal existence. But there are some teeth, the incisors, for instance, of the rodentia, which are capable of indefinite repair. The process of dentition, too, by substituting new teeth for those which are worn out, is a mode employed by Nature for softening, in some measure, the harshness of her own decree.

### III. Of the Human Teeth.

We may disregard the descriptions given both by M. Blandin and by Mr. Hunter of the forms of human teeth, and of their divisions into incisores, molares, and so forth. We shall therefore examine at once:

A. *The Organization of the Teeth.*

M. Blandin examines first the organization of the hard, then that of the follicular part of the teeth.

*a. Organization of the Hard Parts.*—These consist, as our readers well know, of the ivory and the enamel. Bertin and Dr. Emanuel Rousseau, curator of the Museum of Natural History, have asserted that there is a third substance, internal to the innermost layer of ivory, and tending to encroach on the central pulp. M. Blandin, however, rejects this description as inaccurate, and considers this substance, when it apparently exists, as only ivory modified by a papilla not perfectly sound, or an ossification of the papilla itself.

The *ivory* constitutes the bulk of the tooth. We need not dwell on its form and situation. Its density is great. When treated by weak nitric acid, its calcareous matter is removed, and a flexible mass of the form of the ivory remains, which is capable of being reduced to gelatine by boiling. When calcined, it blackens, and leaves a friable residue. Thus the analogy between ivory and bone is perfect.

According to Berzelius, 100 parts of ivory are composed of:

| | |
|---|---|
| Phosphate of lime . . . . . | 61.95 |
| Fluate of lime . . . . . . | 2.10 |
| Phosphate of magnesia . . . . | 1.05 |
| Carbonate of magnesia . . . . | 5.30 |
| Soda, and chloride of sodium . . | 1.40 |
| Animal matter and water . . . | 28.00. |

According to Pepys, 100 parts of the roots of teeth are composed of:

| | |
|---|---|
| Phosphate of lime . . . . . . . | 58.0 |
| Carbonate of lime . . . . . . . | 4.0 |
| Animal matter . . . . . . . . | 28.0 |
| Water and loss . . . . . . . | 10.0. |

We may observe that Berzelius and Morichini only have succeeded in discovering fluate of lime in the ivory. Fourcroy, Vauquelin, Wollaston, and Brandt, could not detect it.

*Enamel.*—While most anatomists confine the enamel to the crown, Bertin affirms that it forms a thin layer on the whole surface of the root. The latter opinion we believe to be unfounded. We shall not speak of the form or of the physical structure of the enamel. They must be familiar. Its chemical composition, according to Berzelius, is as follows. In one hundred parts, there are of:

| | |
|---|---|
| Phosphate of lime . . . . . . . | 85.3 |
| Fluate of lime . . . . . . . . | 3.2 |
| Carbonate of lime . . . . . . . | 8. |
| Phosphate of magnesia . . . . | 1.5 |
| Soda and muriate of soda . . . | 1. |
| Animal matter and water . . . | 1. |

M. Blandin subjoins a pretended account of the analysis of Berzelius, which is so grossly and palpably incorrect, that we are astonished at its remaining in the text.

According to Pepys, 100 parts contain:—

| | |
|---|---|
| Phosphate of lime . . . . . . . | 78.0 |
| Carbonate of lime . . . . . . . | 6.0 |
| Water and loss . . . . . . . . | 16.0. |

If the analysis of Berzelius be correct the enamel differs from the ivory, in having much less animal and more earthy matter. Many persons suppose that there is no animal matter in enamel. This would seem to be an error.

Between the enamel and the ivory, there may be seen a greyish line, which Cuvier has accurately described. This line ends at the neck of the tooth, and is continuous with that part of the follicle which adheres to the root of the tooth. This line marks the existence of a membrane. Before the ivory is formed, this membrane invests the pulp closely. As the ivory is secreted the pulp retires and shrinks, and then the ivory intervenes between the pulp and the membrane. Upon the latter, the capsule forms the enamel, and the membrane being compressed between the enamel and the ivory, all that is at length perceptible is the greyish line to which reference has been already made. This line or membrane, however, if M. Blandin may be trusted, serves as a connecting medium between the hard parts of the tooth and the follicle.

M. Blandin draws two analogies. The first compares the disposition of the ivory and the enamel of the tooth with the bone and articular cartilage of a diarthrodial joint. And a faint analogy both of structure and function is certainly perceptible.

The second analogy is between the ivory of teeth and the horny matter of the tegument, for example hair. This analogy was first announced by Aristotle. The ivory, like the hair, is arranged in concentric layers, and grows upon a vascular pulp.

*b. Organization of the Follicle.*—All that Mr. Hunter and his editor, Mr. Bell, have to say on this point may be dispatched so readily that we quote the passage written by the one, and the marginal note of the other. Mr. Hunter speaks of the follicle under the name of the *Periosteum of the Teeth.* He observes:—

"The teeth, as we have observed, are covered by an enamel only at their bodies; but at their fangs they have a periosteum, which, though very thin, is vascular, and appears to be common to the tooth which it incloses, and the socket, which it lines as an investing internal membrane. It covers the tooth a little beyond the bony socket, and is there attached to the gum." 20.

Mr. Bell corrects him thus:—

"The periosteum can hardly be said to be common to the tooth and the socket. It is, in fact, continued from the external surface of the alveolar process into the socket, and then reflected over the surface of the root. Thus, if a healthy tooth be removed from a dead body, it will be found covered with an extremely thin periosteum, and the alveolar cavity will also often remain lined by a similar structure. Probably when inflammation has once existed to a considerable degree in this membrane, the two layers become permanently united." 20.

We turn to M. Blandin. He thus describes the alveolo-dentar periosteum of anatomists, under the designation of the dental follicle.

The dental follicle, he says, locked in between the root of the tooth and the sides of the alveolus in the adult, is so compressed and adherent to both, that it is distinguished with difficulty in parts of its extent. Still its continuity with the mucous membrane of the gum is manifest. It is formed of two membranous layers—one external, of a fibrous character, confounded with the proper periosteum of the alveolus;—the other internal, more vascular, and adherent to the root of the tooth, up to its neck. In extracting a tooth the internal layer is occasionally separately removed along with it, the external being left attached to the socket.

The *pulp* of the follicle is evidently formed essentially of the dental vessels and nerves in a pretty equal proportion.

Do these vessels and nerves extend beyond the pulp? In other words, has the ivory a vascular organization? This is a question which has long divided the anatomical world. We shall not attempt to go fully into it, for that would occupy some space, but we shall simply state the leading facts and conclusions of Hunter, Bell, and Blandin.

Setting aside the admitted fact, that the teeth cannot be injected artificially, Mr. Hunter arrived at the conclusion, that the ivory of teeth has no vascular organization, chiefly from the results of his experiments with madder.

"Take," he says, "any young animal, as a pig, and feed it with madder for three or four weeks; then kill the animal, and upon examination you will find the following appearances: first, if this animal had some parts of its teeth formed before the feeding with madder, those parts will be known by their remaining of the natural colour; but such parts of the teeth as were formed while the animal was taking the madder will be found to be of a red colour. This shows that it is only those parts that were formed while the animal was taking the madder that are dyed; for what were already formed will not be found in the least tinged. This is different in all other bones, for we know that any part of a bone which is already formed is capable of being dyed with madder, though not so fast as the part that is forming; therefore, as we know that all other bones when formed are vascular, and are thence susceptible of the dye, we may readily suppose that the teeth are not vascular, because they are not susceptible of it after being once formed. But we shall carry this still further: if you feed a pig with madder for some time, and then leave it off for a considerable time before you kill the animal, you will find the above appearances still subsisting, with this addition, that all the parts of the teeth which were formed after leaving off feeding with the madder will be white. Here then in some teeth we shall have white, then red, and then white again; and so we shall have the red and the white colour alternately through the whole tooth.

This experiment shows that the tooth, once tinged, does not lose its colour: now as all other bones that have been tinged lose their colour in time, when the animal leaves off feeding with madder (though very slowly), and as that dye must be taken into the constitution by the absorbents, it would seem that the teeth are without absorbents as well as other vessels." 18.

Accordingly Mr. Hunter considered teeth as "extraneous bodies with respect to a circulation through their substance." But he also considered them to have "a living principle," or they would not be capable of uniting with any part of a living body.

Mr. Bell is a decided advocate of the vitality of teeth, and, as he brings forward the strongest facts that we ever remember to have seen adduced in its favour, we shall extract his note upon the subject.

"The failure of all attempts to inject the substance of the teeth, with even the finest of the matters usually employed for filling the minute branches of arteries in other structures of the body, can scarcely be considered as conclusive, since the colouring matter is, in all of them, too dense and coarse to pass into the vessels of many other parts, which, though in the healthy condition they do not convey red blood, yet, in a state of inflammation, become evidently injected with red particles. I have, however, on another occasion, alluded to two very conclusive facts, which appear unanswerably to prove the vascularity of the teeth. One is the occasional occurrence of red patches in the otherwise healthy bony structure of a tooth when much inflamed, discoverable by breaking or sawing asunder the body of the tooth immediately after extraction. The other, the injection of a tooth with bile in cases of jaundice, of which I have seen more than one example. In the former instance, the red patches are of rather a bright colour, until they become dull and obscure by time; and in the latter the whole substance of the tooth is imbued with a bright yellow colour.

The experiments on madder would be far from conclusive on the point at issue, even were the details more complete. In the absence, however, of all information respecting the duration of each experiment, and especially the period which elapsed after the madder had been discontinued before the animal was killed, it would be futile to combat the conclusions which Hunter has deduced from them. Thus deficient, they only prove that the more highly organized bones, as might be expected, lose the colouring matter of the madder by absorption sooner than the teeth. The paragraph in which the results are summed up is perhaps as characteristic of the peculiar tendencies of Hunter's mind as any that can be found in his works. Concluding, from the failure of his experiments with madder, and from other peculiarities, that the teeth are devoid of a circulation through their substance, and therefore to be so far considered as extraneous bodies, his intense love of truth, which never suffered him even to escape from a dilemma by its slightest sacrifice, forces him to confess the existence of a living principle, because he found, from the result of other experiments, that they 'were capable of uniting with any part of a living body.' Instead, therefore, of endeavouring to render his theory consistent with itself, by disguising or perverting one of the two incompatible conclusions to which his observations had forced him, he adopts them both, and is thus driven to the inference, that an organ can, at the same time, be 'an extraneous body,' and yet possess 'a living principle,' and be 'capable of uniting with any part of a living body.'

The truth appears to be, that the teeth are truly organized bodies, having nerves and absorbent and circulating vessels, but possessing so low a degree of living power, and so dense a structure, as to exhibit phenomena, both in their healthy and diseased condition, which are very dissimilar from those which are observed in true osseous structures." 19.

It would be desirable that Mr. Bell's strong facts should be corroborated by other observers. Mr. Bell's evidence is as forcible as the evidence of one man can be, but one witness on a controverted point of science is never deemed sufficient. Our judgment is so apt to be influenced by our opinions, that, insensibly to ourselves, " whatever we think we believe, and whatever we believe we see;" and this it is which has given birth to so many contradictions on questions merely of fact.

We would observe that it is not necessary for a part to be *appreciably* organized, in order that it may remain in contact and connexion with the living tissues. The epidermis, for example, has no sensible organization, and if the horny matter between it and the cutis is vascular at all, its vascularity is of too low a character to be rendered evident to sense. The ivory of a tooth corresponds with the horny matter in question, the enamel with the epidermis. So far as probability, analogy, and evidence can carry us, we may conclude that the organization and vitality of the ivory is superior to that of enamel, and inferior to that of the osseous tissue in general. The exact quantum of organization and vitality it would be difficult, and perhaps not profitable, to determine.

After stating the arguments of the partisans and enemies of the organization of the hard parts of a tooth, M. Blandin appends his own conclusions. They are as follows:—

1. Vessels and nerves do not enter into the structure of either the ivory or the enamel.

2. The ivory enjoys upon its surface a peculiar sensibility, particularly exhibited in certain kinds of caries, which commence between the enamel and ivory in cases where the enamel has been removed by filing, or in other manners.

3. Can it be possible, says M. Blandin, that this sensibility resides in the *grey line*, to which reference has been already made?

4. This supposition will explain the instantaneousness with which toothache follows certain applications, such as acids, to the surface of the tooth. The thin enamel only imbibes and transmits them.

These conclusions appear to us to be rather favourable to the views of those who regard the ivory as organized than otherwise. For the admission of sensibility on its surface is positive, while the theory of the seat of that sensibility in the "grey line" is dubious.

B. *Development of the Teeth.*

This is both a curious and an interesting subject. We remember well the difficulty we experienced as students, in comprehending the mode of growth of teeth, from the descriptions of the lecturer, and indeed from those of the systems of anatomy. Even at the present day, there are perhaps few who rightly comprehend the process. We shall present as succinct an account of it as possible.

*a. Formation and Structure of the Follicle.* If, says M. Blandin, we examine, about the second month of intra-uterine existence, the alveolar arches of the fœtus, we find a great number of dental follicles, lodged in the substance of the membranous folds which form the gum. These follicles are very small, situated in the gutter, which at this early period represents the alveoli, and covered by the deepest layer of the gum. Their form is globular; superiorly they have an union with the gum, and inferiorly with the bottom of the alveolar gutter, and with the vessels and nerves that lie in it below; laterally they are contiguous to the neighbouring follicles; and anteriorly and posteriorly they are bounded again by the gum.

After the fourth month, according to M. Serres, fibrous septa are developed between the follicles; these septa subsequently ossify, and separate of course the rudiments of the teeth from each other.

At the period of birth, the follicles are perfectly insulated from one another, as well as from the dental vessels and nerves; the canal for the latter, which at first was confounded with the alveolar gutter, is completed.

On opening a dental follicle at an early stage of fœtal life, it is found to contain a yellowish, viscid liquid—acid according to some persons, alkaline according to others—and occasionally presenting a fatty appearance. This liquid progressively diminishes in quantity from the first appearance of the tooth to its final exit from the follicle, at which period it disappears altogether.

The bottom of the follicle is occupied by a large papilla, the form of which corresponds with that of the future tooth which it is to form. The opposite or superficial extremity of the follicle is connected with the gum, by means of a prolongation which constitutes the *gubernaculum*, or *iter dentis*.

This *gubernaculum dentis* is the neck of the dental follicle. It is so contracted, as to present no appreciable cavity in the early stages of development, though subsequently the cavity is supposed to dilate for the passage of the tooth. We say supposed, for while some anatomists believe it does dilate, others deny its permeability. Nay, there is a difference of opinion even with respect to its existence, for while some believe that the follicles of both the first and second dentition possess it, others limit it altogether to the latter.

On the whole, two things may be considered certain, and are not in any way disputed;—*that* there is a sac or follicle, formed of a membrane united to the gum; and, *that*, a pulp, or papilla rises from the bottom of this sac into its interior, the papilla being supplied or formed by the dental vessels and nerves which enter at its root.

But the exact composition of the sac or follicle has been and remains contested.

Mr. Hunter's opinions on this subject are briefly but decisively expressed. Speaking of the pulps he remarks:—

"They grow nearly as large as the body of the tooth before the ossification begins, and increase a little for some time after the ossification has begun. They are surrounded by a membrane, which is not connected with them, excepting at their root or surface of adhesion. This membrane adheres by its outer surface all around the bony cavity in the jaw, and also to the gum where it covers the alveoli.

When the pulp is very young, as in the fœtus of six or seven months, this membrane itself is pretty thick and gelatinous. We can examine it best in a new-born child, and we find it made up of two lamellæ, an external and internal: the external is soft and spongy, without any vessels; the other is much firmer and extremely vascular, its vessels coming from those that are going to the pulp of the tooth: it makes a kind of capsule from the pulp and body of the tooth. While the tooth is within the gum there is always a mucilaginous fluid, like the synovia in the joints, between this membrane and the pulp of the tooth.

When the tooth cuts the gum, this membrane likewise is perforated, after which it begins to waste, and is entirely gone by the time the tooth is fully formed, for the lower part of the membrane continues to adhere to the neck of the tooth, which has now risen as high as the edge of the gum." 38.

Thus Mr. Hunter describes the follicle as consisting of two layers, united at first to the base or root of the pulp, and, when the tooth has been formed, to its neck.

Mr. Bell does not quite coincide with Mr. Hunter. His dissent and his views are both expressed in a note which we transcribe.

"The statement that the bone of a tooth is produced from the pulp is erroneous. This substance constitutes only the mould upon which the ossification is formed, between which and the pulp is placed a membrane of extreme tenuity, which I have termed the proper membrane of the pulp. It is slightly attached to the surface of the pulp, which it completely covers, and it is from the outer surface of this membrane that the bone is secreted. As the pulp recedes on the deposit of the successive laminæ of bone, the ossific membrane continues to cover it, and ultimately forms the well-known membrane lining the internal cavity of the perfect tooth.

The double investing membrane or sac which surrounds the whole rudiment as far as the neck of the tooth, and no further, has been proved, by repeated injections, to be vascular throughout, though Hunter states the internal, and Blake the external layer to be exclusively so. It is from the inner surface of this capsule that the enamel is secreted, as will be more particularly noticed hereafter. As soon as the enamel is secreted the capsule becomes absorbed, beginning at the edge or horizontal surface of the tooth, where the enamel is first deposited. It is, therefore, not perforated by the rising of the tooth, as inferred in the text, but absorbed as soon as it has performed its single function." 39.

Jourdain and Herissant believe that the membrane of the sac is a single one, lining the alveolus down to the root of the papilla, at the level of which it terminates.

Desmoulins, Serres, and Cruveilhier believe also that the membrane of the sac is single, but they are of opinion, that, having arrived at the pedicle of the papilla, it is reflected on it to a height which they cannot satisfactorily determine.

Bichat and Cuvier concur in the existence of two laminæ in the follicle, but they affirm that the external lamina terminates on the pedicle of the papilla, while the internal like a serous membrane is reflected over it. According to this view, the follicle would resemble the fibro-serous pericardium, or dura mater and arachnoid.

M. Delabarre is an advocate for the two layers. The external he supposes to proceed from the fibro-cartilaginous tissue which covers the alveoli, and which helps to form the gum, and descends to the pedicle of the papilla, near which it terminates; the internal layer, continuous with the buccal mucous membrane, descends towards the lateral part of the papilla, and terminates at the point which corresponds with the neck of the future tooth.

M. Blandin having enumerated, endeavours with partial success to reconcile these seemingly jarring statements. He thinks that, in many instances, at all events, those who have described two membranes in the follicle, have counted the alveolar periosteum as one of them; while those who have limited the sac to one membrane have reckoned the alveolar periosteum apart. M. Delabarre's view is justly deemed by M. Blandin the least probable of all.

M. Herissant has made some observations on the internal membrane of the sac, which have been, in some degree, confirmed by Rousseau, Desmoulins, and Cruveilhier, and which are, therefore, deserving of attention. The observations are the following:—

If the internal membrane of the follicle is carefully lifted from the ivory crown of the tooth, and its internal surface is instantly examined with a lens of about a quarter of an inch focus, the observer is struck with the appearance of a great multitude of minute vesicles, transparent, and disposed in order in rows, which are mostly parallel to the base of the tooth. At a certain period, these vesicles contain a limpid fluid, which at a later period becomes milky and thickened. M. Herissant conceives that this fluid being poured upon the ivory, and condensing on it, forms the enamel, by a process resembling crystallization.

Such is the anatomical description of the parts which more immediately concur in the formation of the tooth. It would be idle and tedious to enter on the alterations of the form of the jaw, or on other circumstances adapted for the lecture-room, but not for a journal. We shall therefore set these elements of formation to work, and observe the mode in which a tooth is built up by them. In this we shall be brief, and either omit, or pass very lightly over familiar matters.

*b. Formation of the Ivory and Enamel.*—All agree that the papilla forms the ivory. This was anciently, but erroneously, supposed, to be an osseous transformation of the surface of the papilla. It is now admitted to be a true secretion from that surface, whether, as Mr. Bell contends, with the intervention of an investing membrane, or not, we cannot pretend to determine.

The formation of ivory begins at the crown, and at as many points as there will be future *cuspides* upon the tooth. The secretion of the calcareous matter is preceded by an evident redness of the papilla.

The formation of the enamel is not so simple, or, rather, not so well understood, as that of the ivory of teeth.

Some anatomists have insisted that the enamel is formed before the ivory. The general, and to all appearance the correct, opinion is the other way. But Cuvier affirms that both enamel and ivory are formed at the same time.

Hunter's account of the mode in which the enamel is deposited merits the attention of the physiologist.

There is, he says, another pulpy substance opposite to that which we have described: it adheres to the inside of the capsule, where the gum is joined to it, and its opposite surface lies in contact with the basis of the above-described pulp, and afterwards with the new-formed basis of the tooth. Whatever eminences or cavities the one has, the other has the same, but reversed, so that they are moulded exactly to each other.

Mr. Bell, in a note on this section remarks, that the substance which Hunter terms "another pulpy substance," adhering to the inside of the capsule, is nothing more than a thickened and turgid state of the inner layer of the capsule itself, surcharged with blood and probably also with the earthy matter which it is about to deposit, constituting the future enamel-covering of the crown of the tooth. This thickening appears to be somewhat analogous to that extraordinary turgescence which is observable in the mantle of certain species of snail, as our own *Helix Pomatia*, immediately before the calcareous winter operculum is poured out from every part of its surface.

Mr. Hunter continues:—

"After the points of the first-described pulp have begun to ossify, a thin covering of enamel is spread over them, which increases in thickness till some time before the tooth begins to cut the gum.

The enamel appears to be secreted from the pulp above-described, and perhaps from the capsule which incloses the body of the tooth. That it is from the pulp and capsule seems evident in the horse, ass, ox, sheep, &c., therefore we have little reason to doubt of it in the human species. It is a calcareous earth, probably dissolved in the juices of our body, and thrown out from these parts, which act here as a gland. After it is secreted, the earth is attracted by the bony part of the tooth which is already formed, and upon that surface it crystallizes.

The operation is similar to the formation of the shell of the egg, the stone in the kidneys and bladder, and the gall-stone. This accounts for the striated crystallized appearance which the enamel has when broken, and also for the direction of these striæ.

The enamel is thicker at the points and basis than at the neck of the teeth, which may be easily accounted for from its manner of formation; for if we suppose it to be always secreting, and laid equally over the whole surface, as the tooth grows, the first formed will be the thickest; and the neck of the tooth, which is the last-formed part inclosed in this capsule, must have the thinnest coat; and the fang, where the periosteum adheres, and leaves no vacant space, will have none of the enamel.

At its formation it is not very hard; for by exposing a very young tooth to the air the enamel cracks and looks rough; but by the time that the teeth cut the gum, the enamel seems to be as hard as ever it is afterwards; so that the air seems to have no effect in hardening it." 43.

But Cuvier and Serres have strongly opposed these sentiments and descriptions of Hunter. M. Serres contends that the liquid of the follicle has no relation whatever with the formation of the teeth, but that it escapes on the opening of the follicle by the tooth in dentition.

M. Cuvier, whose description of the two laminæ of the follicle may be remembered, or may be ascertained by turning back a page or two, asserts that the enamel is deposited not immediately upon the ivory crown, but upon the internal layer of the follicle, which afterwards forms the greyish line intervening between the ivory and the enamel. M. Cuvier and his school regard the enamel as a true secretion from the interior of the follicle, and the difference between this opinion and Hunter's is, after all, not great. Hunter looked on the fluid as the secretion, and the enamel as crystallized from it. Cuvier regards the enamel as the direct product of secretion.

The reason why the root of the tooth is not enamelled has been differently explained according to the different theories of the formation of the enamel itself.

As, by Cuvier's theory the enamel is secreted in the sac, and the ivory out of it, the crown of the latter merely pushing the internal layer of the sac before it, it is clear that, as the sac does not cover the root, the latter cannot obtain enamel.

If the theory of crystallization is admitted, we may suppose that, the crown having been covered with enamel, the fluid, from which the latter is deposited, is exhausted.

Herissant conceives that the glands he describes as secreting the enamel from the inner surface of the sack, having coated the crown with their secretion, waste, and no more enamel is formed.

It appears to us, that as the parietes of the follicle are generally admitted to adhere to the pulp directly or mediately, opposite the site of the future neck of the tooth—and, as the follicle is generally admitted also to constitute the efficient agent in the formation of enamel, we should naturally expect the latter not to extend beyond the neck of the tooth.

### c. *Growth of the Fang.*

Mr. Hunter's account of the increase of the ivory and formation of the fang is more concise than M. Blandin's, and is quite correct. We therefore turn to our countryman.

When the bone, he says, has covered all the pulp, it begins to contract a little and becomes somewhat rounded, making that part of the tooth which is called the neck; and from this place the fangs begin. When the fangs form, they push up the bodies of the teeth through the sockets, which waste, and afterwards through the gum, which also wastes.

The pulp, he proceeds, has originally no process answering to the fang; but as the cavity in the body of the tooth is filled up by the ossification, the pulp is lengthened into a fang. The fang grows in length and rises higher and higher in the socket till the whole body of the tooth is pushed out. The socket at the same time contracts at its bottom and grasping the neck or beginning fang, adheres to it and rises with it, which contraction is continued through the whole length of the socket as the fang rises: or the socket which contained the body of the tooth, being too large for the fang, is wasted or absorbed into the constitution, and a new alveolar portion is raised with the fang; whence in reality the fang does not sink or descend into the jaw.

As the tooth grows, its cavity, of course, becomes gradually smaller, for the pulp having formed layer within layer of ivory upon its surface, must necessarily have shrunk to allow room for their deposition. Of these lamellæ of the ivory, the outermost, or first formed, is the shortest; the more internal grow successively longer, and form more and more of the length of the fang.

A tooth which has more than one root has a number of processes to its papilla, corresponding to the number of its roots.

As a natural limit to the deposition of the ivory results from its encroachment on the pulp, and the consequent diminution of this, the organ of secretion, so the same diminution or atrophy may be supposed to be the immediate cause of the cessation of the increase in length. Indeed the increase in one sense hinges on the increase in the other. For the innermost layers effected the elongation of the root, and when those layers cease to be formed the tooth must cease to lengthen.

### D. *Dentition.*

We shall not tire our readers by dilating on the phenomena of dentition. We shall merely advert to a theory to which allusion has already been made, that the tooth does not actually produce ulceration of the gum, but traverses an opening ready made for it—the *iter dentis.*

The neck of the follicle has been already described. Some anatomists suppose that the pressure of the growing tooth opens this, and that, by its dilatation, and the ulceration of the false cartilaginous gum, dentition is effected. However plausible this may appear, we must confess that, in the dissections we have made, we have seen nothing that gives it countenance.

### E. *Formation of the Permanent Teeth.*

Mr. Hunter's account of the mode of growth of the permanent teeth is confessedly imperfect, if not actually erroneous. Mr. Bell supplies an account, which we have great pleasure in quoting.

The rudiments of the permanent teeth are a sort of offset from the temporary. They are long connected with the latter, an important fact both practically and physiologically.

"At an early period," says Mr. Bell, "in the formation of the temporary

E 2

teeth, by a process which reminds us of the gemmiparous reproduction in the lower grades, both of animal and vegetable life, the investing sac, or capsule, gives off a small process, a bud containing a portion of the essential rudiments, namely, the pulp, covered by its proper membrane. This constitutes the rudiment of the permanent tooth. It commences in a small thickening on one side of the parent sac, which gradually becomes more and more circumscribed, and at length assumes a distinct form, though still connected with it by a peduncle, which is nothing more than a process of the investing sac. For a time the new rudiment is contained within the same alveolus with its parent, which is excavated by the absorbents for its reception, by a process almost unparalleled in the phenomena of physiology. It is not produced by the pressure of the new rudiment, as has been erroneously believed, but commences in the cancelli of the bone immediately within its smooth surface, thus constituting what may be termed a process of anticipation. The new cell, after being sufficiently excavated, and as the rudiment continues to increase, is gradually separated from the former one, by being more and more deeply excavated in the substance of the bone, and also by the deposition of a bony partition between them ; and at length the new rudiment is shut up in its proper socket, though still connected with the temporary tooth by the cord or process of the capsule already described, which has in the mean time been gradually attenuated and elongated.

The situation of each permanent rudiment when its corresponding temporary tooth has made its appearance through the gum, is beneath and a little behind the latter, and rather further from the centre of the jaw. From the preceding statement, then, it will be readily understood that the upper part of the new sac being, by means of the cord, connected with the gum, assumes, by-and-by, the same relation to that substance as that which the temporary rudiment, as before described, had originally sustained; whilst, from its substance being imbedded in the jaw, the vessels and nerves which had entered into the composition of the new process of pulp in its first production, probably became so enlarged and modified in their structure as ultimately to form the true dental branches. This is much more probable than to suppose that a new set of nerves and vessels is given off from the maxillary branches to join the pulps at a distance, through an intervening layer of bone of an indefinite thickness, to supply every new tooth.

We now, therefore, find the new rudiment in a state nearly analogous to that in which the parent tooth was originally placed, and with similar relations to the surrounding parts; the sac above, attached to the gum, and the pulp beneath, (covered with its proper membrane,) connected by its vessels, &c. with the jaws." 38.

The descriptions of M. Blandin do not coincide, in all respects, with the one we have quoted from the notes of Mr. Bell. We will present a brief summary of the former.

The germs of the permanent teeth may be perceived about the time when those of the temporary teeth are evident—about the third month of fœtal life. They are very small, suspended from the membrane of the gum by a mucous filament about a line in length, and situated behind the germs of the first dentition. With the growth of the jaw, the germs of the permanent teeth descend (in the lower jaw) from the same horizontal plane with the germs of the temporary teeth, and are placed below them. This change of situation is permitted by a remarkable elongation of the "mucous filament," "neck," or "ductus dentis."

In the first instance, the germs of the two dentitions are contained in the same alveolus, but, at a later period, partitions are developed, and constitute distinct alveoli for each dentition. This happens about a year after birth, when the partitions are ossified.

The alveoli of the permanent germs are pierced by the opening of a canal at each extremity—below, for the vessels and nerves of the pulp—above, for the "neck" or "ductus" of the follicle. The canals for the ductus do *not* open, according to the observations of M. Blandin, into the alveolus of the temporary tooth, but terminate on the alveolar border, behind the alveolus in question.

The follicle of the permanent teeth is a dependence of the mucous membrane of the mouth, as that of the temporary teeth is. The ductus traverses the canal appropriated to it, in order that it may reach that membrane, or, rather the canal has grown to accommodate it. Meckel has advocated the doctrine taken up by Mr. Bell, and quoted in these pages from him, that the germ of the permanent tooth is a sort of gemmiparous production from that of the temporary tooth ; but M. Blandin disputes this, for he observes that the communication between them is only by the external membrane of the follicle, while the internal membrane of the respective follicles, is distinct in each. As the internal membrane is the efficient agent in the deposition or formation of the tooth, this appears to be a strong objection to the view ; and indeed when we reflect that the number of the permanent teeth much exceeds that of the temporary, and that, consequently, many of the germs of the former cannot enjoy the benefit of parents in the latter, we may conclude that the idea of Meckel is rather fanciful than just. In all probability the connexion between the germs of the two sets, is rather for the convenience of furnishing an early vascular supply to the permanent germs, and, at a later period, of determining their situation, than for any more recondite purpose.

We should observe that the dental canal, for lodging the trunks of the vessels of the temporary germs and teeth, is itself temporary, and superseded by the true dental canal for the permanent teeth when they have become developed.

We have now gone into the interesting, yet rather perplexing subject of the mode of formation of the teeth, as deeply as the nature of this Journal, and other considerations, seem to render it advisable to enter. We refer those who wish for more explicit information to the works of Mr. Hunter and of M. Blandin. Of the former it is not necessary to speak. Its value and its defects have been long agreed on. The work of M. Blandin, as it is the last, so it is the most complete and circumstantial, and it presents the best text-book, for the anatomical student and teacher. Its merits are considerable—its faults are chiefly to be found in that redundance of language, repetition of facts, and prolixity of style, which form the too general characteristics of continental medical literature.

But we cannot terminate this article, without presenting a short account of the development of teeth in the ascending scale of organization. Views of this description enlarge our ideas, and give them both generality and precision. Such a view is offered by M. Blandin in the concluding portion of his work, and we shall combine with his observations those of such other comparative anatomists as we find it advisable to introduce.

1. *Teeth of the Invertebrata.*

Many of the invertebrated animals are provided with what serve the offices of teeth, but differ widely from the teeth of the higher classes in structure.

These pseudo-teeth, or dentiform organs of the invertebrata, are placed at the entrance, or near the entrance, of the alimentary canal, and serve to seize, retain, or comminute the alimentary substances. The lower the animal in the scale, the greater the probability that these teeth will be found in the stomach, rather than nearer the buccal orifice. Thus the crustacea and mollusca have principally these stomachic teeth—in fish they are not found lower than the pharynx—in reptiles they are seen in the posterior part of the mouth—and, in mammalia, they are placed on its anterior and lateral parts.

Insects and worms can scarcely be said to possess true teeth. At the entrance of the mouth, the integument is condensed, and the epithelium seems to acquire a horny consistence. Among the radiata, are some instances in which a dental apparatus has been described as existing. In other insects, some of the orthoptera, the locusts, &c., a triturating apparatus, consisting of scales or horny hooks, has been discovered in the stomach.

In crustacea and mollusca, the gastric teeth are peculiarly developed.

The stomach of the crab and lobster is a very singular organ. It is formed on a bony apparatus, in short a species of skeleton ; and does not therefore collapse when empty. To certain parts of this bony structure, round the pylorus, the teeth are affixed. Their substance is extremely hard, and their margin is serrated or denticulated : as they surround the tube, near the pylorus, nothing can pass that opening without being perfectly comminuted. These bones and teeth are moved by particular muscles.

### 2. *Teeth of the Vertebrata.*

*a. Fish.*—The teeth of fish present great varieties in number as well as in the disposition of the parts composing them. They are found on all parts of the internal surface of the mouth and pharynx, and there are some productions of the integument in the neighbourhood, which M. Blainville has been led to regard as true teeth.

In the osseous fish, the teeth are implanted in particular alveoli. In the cartilaginous fish, with the exception of the sword-fish, they are placed at a distance from the bones, which, consequently, cannot supply them with alveoli. The former description of teeth are fixed—the latter are moveable, and capable of erection and depression.

The circumstance of teeth growing in this manner from the mucous membrane, independently altogether of the bones, is a happy illustration of the theory which represents the teeth as simple productions of the tegumentary tissue. The analogy between the teeth of man and the tegumentary teeth of animals lower in the scale is satisfactorily enough established.

In form, the teeth of fish exhibit many variations. Some are very pointed. These are very numerous when they exist, and the points may be single, or double, or triple. These teeth are directed inwards and backwards, so as to obstruct the egress, and facilitate the ingress of the prey.

Other teeth are flattened, and form a sort of pavement. We need not insist upon their various arrangements. We may cite a note from Coulson's Blumenbach.

" A third kind of teeth consists of an assemblage of tubes, covered externally by enamel, and connected to the jaw by a softer substance, which probably sends processes or vessels into those bony tubes. This is the case with the pavement, as we may call it, of teeth, that covers the jaws of the *skate*.

A similar structure is observed in the *anarrichas lupus;* where the teeth, composed of bony tubes, are connected to spongy eminences of the jaws, which may be compared to epiphyses; and on their separation leave a surface like that from which the antler of the deer falls off." 77.

The alveolar teeth of the osseous fishes, become, after a time, anchylosed to the edge of the alveoli. After a variable time, too, they are replaced by other teeth in a manner which is not well understood.

In most of the sharks, says M. Blumenbach, the mouth is furnished with very numerous teeth for the supply of such as may be lost. The *white shark* has more than two hundred, lying on each other in rows, almost like the leaves of an artichoke. Those only, which form the front row, have a perpendicular direction, and are completely uncovered. Those of the subsequent rows are, on the contrary, smaller, have their points turned backwards, and are covered with a kind of gum. These come through the covering substance, and pass forward when any teeth of the front row are lost.

M. Blandin observes that we may readily determine from the nature and disposition of the teeth, the habits of the fish. Has he numerous pointed teeth turned backwards? He is one of the most carnivorous, voracious, and terrible tenants of the ocean. Do his teeth form a sort of pavement in his mouth? he lives on shell-fish, and breaks their shells with his natural nut-crackers. Has he simple teeth only in the mouth or in the pharynx? he is among the most guileless and least carnivorous of his order.

*b. Reptiles* appear to exhibit, in their teeth, their position in the zoological scale, between fishes and birds. The tortoise, on the one hand, has a beak, like the bird; and, on the other hand, many reptiles resemble fish in the number, form, and main characteristics of teeth.

The beak of the chelonian reptile often presents, like that of the bird, dentations, which are a sort of rudimentary teeth. Beneath the horny layer is an osseous plate, applied, but not implanted on the jaws. This plate represents, or is supposed to represent, the ivory of a series of teeth confounded and consolidated together, whilst the horny layer is the analogue of the enamel.

Setting aside the chelonian, the other reptiles have pointed and conical teeth, essentially carnivorous, and not adapted for trituration. On this account they are generally directed backwards, and hooked with the concavity turned backwards too. The number of their teeth is considerable, and not always clearly determined. They are usually attached to the jaws, though in the majority of serpents they are also found on the palate.

The teeth of reptiles belong to the class of teeth without roots. They are conical, hollowed by a papillary cavity of the same form, and the maxillary teeth are lodged in alveoli which are narrower at their aperture than at their bottom; the base of the tooth corresponding with the latter, it is, of course, very firmly fixed in the jaw. The teeth closely resemble one another, and have more of the canine than of any other character.

They are in general developed at a very early period, especially in crocodiles, the young of which has as many teeth at birth, as when the creature is twenty feet long. The teeth grow, as in man, by depositions on the papilla.

The tooth being conical with its base on the papilla would grow indefinitely, but that it is hampered by the narrowness of the orifice of the alveolus.

The teeth of reptiles appear to be regenerated with facility. At whatever age they are pulled out in the crocodile, there are always found beneath or within them the germs of their successors.

We cannot enter on the details of the dentition of reptiles. We shall merely allude to the poison-fang, found in some species of snake. Blumenbach simply states that:—The external row of teeth is wanting in the poisonous species; which have, in their stead, much larger tubular fangs connected with the poison bladder, and constituting, in reality, bony excretory ducts, which convey the venom into the wound, inflicted by the bite of the animal.

M. Blandin remarks that the poison-fangs are semicircular in form, with their concavity turned backwards, and are attached to the superior maxillary bone. They usually occupy the anterior part of the arch of the palate, but in some serpents they are behind. They are not moveable, but the superior maxillary bone is peculiarly so. They are longer than the other teeth, and traversed by a canal; which, at the root of the tooth, receives the excretory duct of the poison gland, while it opens at the point of the tooth, and so carries the venom into the wound. The poison gland itself is the analogue of the parotid, and situate below and behind the orbit on each side. It contains a wide cavity, is embraced by a part of the temporal muscle, which compresses, and can empty it, and its long duct goes to the perforated base of the poison-fang.

In the membranous pouch, which encircles the base of the poison-fang a certain number of rudimentary fangs may be found—sometimes as many as eleven. They are formed in membranous capsules, disposed parallel to one another in the substance of the palatine membrane. Their magnitude is proportioned to their proximity to the fang actually in use. When this falls out, the one that is to replace it, and whose base has hitherto continued membranous, becomes united so accurately to the site of its predecessor, that the opening in its canal corresponds exactly to the duct of the poison gland.

c. *Birds.* We shall say nothing of the beaks and bills of birds—the analogues of teeth—as we quoted in our last number a pretty full account of them from Dr. Grant.

d. *Mammalia.* The subject of the varieties of teeth in the mammalia is too extensive for us to do justice to it here. This article has already extended too far to allow us to add much to what has gone before.

The teeth of mammalia have been divided into simple—composite—and mixed, or intermediate between those two.

The simple teeth are like those of man.

The composite teeth present so many irregularities upon their surface, that they seem formed of several joined together. On sawing them, their component elements are found so disposed that, instead of one being simply external to the other, the saw several times traverses both. They are formed by a number of papillary processes set upon a common base.

The mixed or intermediate teeth are those in which the interstices, &c. penetrate only to a certain depth, and in which the base is simple.

Although the majority of teeth have roots, yet, in some animals, as in the rodentia, and in the tusks of the hippopotamus and elephant, there is no root. A tooth of this description has a conical form, and a great length, the base of the cone being implanted in the follicle.

In most instances, the teeth, as in the human subject, are formed only of enamel and ivory. But the composite and mixed teeth contain a third substance—the crusta petrosa, or cement. By analysis, the latter was found, in one instance, to be composed of—

| | |
|---|---|
| Animal matter . . . . . . . . | 43·01 |
| Phosphate of lime . . . . . . | 52·94 |
| Carbonate of lime . . . . . . | 4·05. |

We extract from Coulson's Blumenbach a brief note, on the arrangement of the component parts of the teeth in the carnivora and some of the granivora.

"All the teeth of the *carnivora,* and the incisors of the *ruminating animals,* have the crown only covered with enamel, as in the human subject. The immense fossil grinders of the *animal incognitum,* or *mammoth,* have a similar distribution of this substance.

The grinders of graminivorous quadrupeds, and the incisors also of the horse, have processes of enamel descending into the substance of the tooth. These organs have also in the last-mentioned animals a third component part, differing in appearance from both the others, but resembling the bone more than the enamel. Blake has distinguished this by the name of *crusta petrosa;* and Cuvier calls it *cement.*

The physiological explanation of this difference in structure is a very easy and clear one. The food of the *carnivora* requires very little comminution before it enters the stomach: hence, the form of their molar teeth is by no means calculated for grinding; and, as the articulation of the jaw admits no lateral motion, these teeth, of which the lower are overlapped by the upper, can only act like the incisors of other animals. The food of graminivorous quadrupeds is subject to a long process of mastication, before it is exposed to the action of the stomach. The teeth of the animals suffer great attrition during this time, and would be worn down very rapidly but for the enamel which is intermixed with their substance. As this part is harder than the other constituents of the teeth, it resists the attrition longer, and presents the appearance of prominent ridges on the worn surface, by which the grinding of the food is much facilitated.

The distinction of the three substances is seen better in the tooth of the elephant than in any animal. The best method of displaying it is by making a longitudinal vertical section, and polishing the cut surface. The *crusta petrosa* will then be distinguished by a greater yellowness and opacity in its colour; and by an uniformity in its appearance, as no laminæ or fibres can be distinguished." 36.

The consequences of the descent of enamel between vertical processes of ivory, and the interposition of processes of cement or crusta petrosa between those of enamel, are obvious on a glance at the grinding tooth of the elephant. For, as the surface of the tooth is worn down in mastication, the processes of enamel, which are capable of making a resistance by their superior hardness, form prominent ridges on the grinding surface, which must adapt it excellently for bruising and comminuting any hard substance.

It is curious that a great controversy raged in the time of Sir Everard Home, on the comparative hardness of the ivory and of the crusta petrosa. One would imagine that a physical fact like this would be susceptible of determination without difficulty. The difference, however, between the ivory and crusta petrosa, in point of hardness, is so slight that, when we endeavour to ascertain the amount of difference between them, we do not find it easy to make up our minds.

There is another provision assuring to a great extent the permanency of the grinding teeth in the elephant, and, indeed, in other animals to whom the grinders are of great consequence. It is thus succinctly described in the notes to Coulson's Blumenbach.

We never see more than one grinder and part of another through the gum in the elephant. The anterior one is gradually worn away by mastication; its fangs and alveolus are then absorbed: the posterior tooth coming forwards to supply its place. As this goes through the same stages as the preceding grinder, a third tooth, which was contained in the back of the jaw, appears through the gum, and advances in proportion as the destruction and absorption of the other proceed. The same process is repeated at least eight times, and each new grinder is larger than that which came before it. The 1st, or milk grinder, is composed of four transverse plates or denticuli, and cuts the gum soon after birth. The 2d, which has eight or nine plates, has completely appeared at the age of two years. The 3rd, formed of twelve or thirteen, at six years. From the 4th to the 8th grinder, the number of plates varies from fifteen to twenty-three, which is the largest hitherto ascertained. The exact age at which each of these is completed has not yet been made out: but it appears that every new one takes at least a year more for its formation than its predecessor.

From the gradual manner in which the tooth advances, it is manifest that a small portion of it only can penetrate the gum at once. A grinder, consisting of twelve or fourteen plates, has two or three of these through the gum, whilst the others are imbedded in the jaw. The formation of the tooth is complete therefore, first, at its anterior part, which is employed in mastication, while the back part is very incomplete; as the succeeding laminæ advance through the gum, their formation is successively perfected. But the posterior layers of the tooth are not employed in mastication, until the anterior ones have been worn down to the very fang, which begins to be absorbed.

But there is a class of animals—the rodentia—the nature of whose habits and food requires cutting teeth, while their permanency is absolutely essential.

The incisor teeth of the rodentia are four in number—two in the upper jaw and two in the lower. They are long, strong, curved with the concavity directed backwards, and covered with enamel almost exclusively on the anterior surface. The consequence of this disposition of the enamel is, that the ivory behind wearing away faster than the enamel in the front, the edge of the tooth is always sharp.

The incisor teeth of the rodentia have no root. The base of the tooth, its broadest part, is set upon the broad and conical pulp, which not being hemmed in by its own secretion continues to secrete indefinitely, and always to form fresh teeth below, as its free extremity is worn away by attrition above. This is the case with the tusks of the hippopotamus and elephant, &c.—the case, in fact, with all those teeth which have no true contracted roots.

A remarkable instance of the tendency to growth of teeth of this description, was presented by M. Devergie to the Academy of Medicine in 1825. It was seen in an old rat, killed at the Ecole Militaire. The right superior incisor tooth, on rising from its alveolus, was curved backwards and downwards so as to enter the mouth, passed through the posterior nares into the left nasal fossa, traversed it from behind forwards, then penetrated the maxillary bone, passed through the left incisive alveolus by the side of the left incisor tooth, was again curved backwards and downwards, and terminated just below the left orbit.

The left superior incisor was equally long, and curved, but did not take a similar direction.

The incisors of the lower jaw were very long, and curved. The right, which was the longer, described almost a complete circle, with a diameter of about eight lines. It passed in front of the orbit, the inferior border of which it had hollowed into a gutter, obliterated its cavity, and the corresponding eye, and its curved point would shortly have entered the cranium.

It is evident that some circumstances must have interfered with the use of these teeth at some period. Their free edges not being worn, and the papillæ constantly adding to their bases, their growth became excessive and their direction as whimsical as we have described it.

Here we must close the present article. The space allotted to us forbade our presenting a complete history either of the human teeth, or of those of other animals. But we have selected some of the most important, or most interesting matters connected with the teeth, both of man and of creatures lower in the scale, and laid them before our readers, in a manner as little disjointed as possible. What we have written may, if attentively perused, both instruct and entertain the less scientific portion of them.

Of M. Blandin it is not necessary again to say much. We recommend his work to those who are anxious to become acquainted with the details of dental anatomy. They will find in it much valuable information, and particulars which they will hardly be able to glean from any other monograph.

---

